# The end of COVID-19: not with a bang but a whimper

**DOI:** 10.1007/s11845-023-03435-1

**Published:** 2023-06-29

**Authors:** Laura Bond, Fiona McNicholas

**Affiliations:** 1 School of Medicine, University College Dublin, Dublin, Ireland; 2 Department of Psychiatry, Children’s Hospital Ireland at Crumlin, Dublin, Ireland

**Keywords:** COVID-19, End, Pandemic preparedness, Restrictions

## Abstract

The formal announcement of the end of the COVID-19 pandemic by the WHO came on the 5th of May 2023; however, unlike the pandemic onset, the pandemic end date was not met with any significant media coverage or news reporting in Ireland. Additionally, there were no series of contemplations either in newspapers or other media about the impact of the decision to formally end the pandemic despite having financial and legislative impacts on a wide number of people. Given the potential impact of the removal of government subsidies on health and occupations, government and media coverage of the decisions and potential implications would have been helpful. The opportunity for a significant debriefing of the pandemic outlining what we have learned from the COVID-19 pandemic response may have been missed.

## The start and end of the COVID-19 pandemic

On the 30th of January 2020, the World Health Organization (WHO) declared a public health emergency of international concern over the global outbreak of coronavirus disease 2019 (COVID-19) [1]. Extensive media coverage followed in Ireland and audiences watched live press conferences for regular updates with government and public health officials daily. News outlets and an innovative COVID-19 tracker app provided detailed reporting of every aspect of the pandemic particularly the government-mandated COVID-19 restrictions as well as daily infection and death rates. COVID-19 restrictions waxed and waned depending on the perceived virulence threat; however, over time they were gradually reduced. By the 22nd of January 2022, most of the major restrictions had been removed with the gradual recognition that the pandemic was going to be endemic [2]. The formal announcement of the end of the pandemic by the WHO came on the 5th of May 2023 [3]; however, unlike the pandemic onset, the pandemic end date was not met with any significant media coverage or news reporting in Ireland. Additionally, there were no series of contemplations either in newspapers or other media about the impact of the decision to formally end the pandemic despite having financial and legislative impacts on a wide number of people.

Reflecting on the 1191 days of COVID-19, the negative impact of the pandemic in Ireland has been significant. According to the WHO, there has been more than 1,711,200 cumulative cases and more than 8900 deaths to date [4]. Deaths are still occurring, although a significantly lower rate prior to January 2021 [5]. The huge strain on healthcare settings linked with delays in elective procedures and non-COVID-related appointments including cancer screening and care has been noted [5]. An increase in mental health difficulties has also been reported since the start of the COVID-19 pandemic, likely attributed to a combination of anxiety around COVID-19 and the effects of lockdown [6]. Additionally, the economic costs from the public health measures have been recognised. Initially, there was a significant retraction of the Irish economy by 6.1% between April and June 2020, which was linked to the pandemic, and an announcement of a recession was made in September 2020 [7]. The closing of businesses resulted in the largest monthly increase of unemployment in history [8], and monthly unemployment rates peaked in March 2021 to 7.7% [9]. Government financial supports were implemented to support businesses and affected workers including the Pandemic Unemployment Payment (PUP) and Employment Wage Subsidy Scheme (EWSS) [5]. The economy has since gradually improved and last year Ireland’s economy was the fasting growing in Europe [10].

## Positive outcomes of the COVID-19 pandemic

Despite the devastating effects of the pandemic, there were several unexpected positive outcomes, which some may wish to hold onto. Working from home improved flexibility and work-life balance, improved finances from petrol savings, reduced the time and stress of commuting and reduced travel emissions [11]. Lockdowns often meant an increase in time spent with family and outside in nature. Whilst this may not be positive for all, for many it provided unique opportunities to rediscover simple pleasures such as family walks, connecting to one another and making memories. Many children and young people reported improved mental well-being during the first lockdown in the United Kingdom (UK) due to improved relationships with friends and family, less loneliness and exclusion, reduced bullying, better management of school tasks and improved sleep and exercise [12].

Adapting to COVID-19 restrictions resulted in significant technological advancements particularly in healthcare and educational settings. In Ireland, there has been a five-fold increase in the use of telemedicine since the start of the pandemic including online consultations and electronic prescribing [13]. Patients have reported high rates of satisfaction due to convenience, ease of access to healthcare professionals, ease of obtaining repeat prescriptions and cost-effectiveness [13]. The expansion of distance learning options has provided convenient, affordable and flexible access to education for adults that may have time, financial or work constraints. The pandemic accelerated advances in vaccine technology and production which has opened the possibility that science could develop new vaccines to combat other diseases including infectious diseases and cancer [14].

In the UK, the Zoe Health Study developed an app in 2020, which collected real-time COVID-19 data so that epidemiological results could be calculated [15]. During the pandemic 4.7 million users downloaded the app and contributed to data collection. Over fifty scientific papers have since been published reporting novel findings around symptoms and vaccine effectiveness [16]. In 2022, the app broadened data to be collected on other diseases such as cancer, heart disease and mental health disorders [16]. The Health Service Executive (HSE) also developed a real-time COVID-19 tracker app where the focus was on contact tracing; however, only 4% of individuals who tested positive during the pandemic used the app for the purpose it was intended [17].

## Implications of ending the COVID-19 pandemic

The exact implications of formally ending the pandemic are significant for many but have been less well discussed. During the pandemic in the United States (US), the federal government required states to continuously enrol Medicaid recipients into the programme, enhancing health insurance coverage to adults and children with limited income and resources [18]. There is a concern that when the pandemic health emergency declaration expires in July 2023 up to 14.2 million people could lose their Medicaid coverage and already hundreds of thousands of low-income Americans have lost their coverage [18].

In Ireland, there has been a loss of financial supports such as the PUP and EWSS, which ended in March and May 2022 respectively [19]. Older workers (+ 55) who were made unemployed were reported to be the most likely to struggle in getting back into work with hesitancy over health concerns and lack of technological resources and skills as being possible explanatory factors [20]. Although employment rates have recovered to pre-pandemic levels, some sectors and occupations have never returned or continued to decline as the economy adjusts to a post-pandemic world [21]. Whilst some can opt to retrain or upskill to seek alternative employment, others including non-Irish nationals and lower skilled workers may not have access or financial means to the same opportunities [22]. Therefore, the loss of the COVID-19 financial supports will likely have an adverse impact on these vulnerable cohorts.

The Enhanced Illness Benefit was introduced at the start of the pandemic as a payment for employees or those self-employed required to isolate due to COVID-19 at a weekly rate of €350 [23]. The scheme was terminated in September 2022 [23]. In comparison to the standard illness benefit, the weekly rate was higher and started immediately compared to 3 days later. The aim was to incentivise people to self-isolate if they had COVID-19 or suspected COVID-19 to reduce transmission. With the loss of this scheme and incentivisation, there is an increased risk of potentially infectious people returning to work and increasing transmission to other more vulnerable work colleagues. Additionally, although people can still avail of the standard illness benefit, the lower payment rate may not provide the required support.

The HSE’s COVID-19 testing centres, which provided free COVID-19 PCR tests, were closed in March 2023. There is now a cost to be civic-minded as COVID-19 antigen tests must be purchased in pharmacies or shops or organised through a person’s GP with private patients required to pay. At the start of the COVID-19 vaccine rollout, Ireland’s vaccine uptake was reported to be amongst the most successful globally and ranked first in the European Union (EU) in terms of percentage of the adult population fully vaccinated [24]. More recently, there has been a sharp decline in the uptake of COVID-19 booster vaccines with just 26% of eligible adults receiving a second booster since January 2023, which is significantly below the HSE’s target of 75% [25]. The public may no longer perceive COVID-19 as a serious threat and thus are less inclined to make appointments and travel for vaccination. The lower than hoped for booster uptake and ongoing COVID-19 cases suggests that the need for testing remains. There is a concern that the financial cost of COVID-19 tests plus the loss of benefits afforded if one needs to sick leave may reduce the number of people voluntarily testing.

There are several pandemic-related changes which may remain for the future. Working from home either full term or as part of a hybrid option is likely to remain given its popularity. The job website “Indeed” reported that searches for jobs in Ireland that allow remote working were six times higher compared to 2019 [26]. This may have a positive impact on Ireland’s housing crisis as people are able to live rurally rather than concentrating in urban areas; however, this will require an equitable broadband rollout. Many health and adult educational settings, whilst resuming traditional in-person services, still offer telemedicine and distance learning options. Videoconferencing in occupational settings largely remains given its convenience.

COVID-19 has resulted in the recognition that presenteeism negatively impacts both the person working when they are ill with regard to their own mental health and on others who are at risk of infection. The recommendations and application of people staying at home when infected with COVID-19 until they were no longer able to transmit the infection were a positive public health benefit. Whether this practice will be maintained going forwards will depend on whether the government and employers promote it positively and support it financially. Similarly, although mask wearing has been formally phased out, there is a public health benefit of people being civic-minded and opting to wear masks if they are unwell to protect others or if visiting clinically vulnerable people. This approach was already ingrained in many Asian cultures prior to the pandemic, and it may become a permanent behavioural change globally.

## Future pandemic preparedness

COVID-19 has highlighted the necessity of future pandemic preparedness. One of the critical issues in Ireland during COVID-19 was the low number of ICU beds in public hospitals (5.5 per 100,000 compared to the European average of 12 per 100,000) [27]. The Irish government responded quickly and appropriately by incorporating the private hospitals into the public health system and increasing the public ICU beds. However, it is recognised that staffing and recruitment continue to be ongoing stressors for the Irish health system. High levels of burnout, job dissatisfaction and mental ill health amongst healthcare staff have been highlighted as explanatory factors for recruitment and retention issues [28].

It is now recognised that some of the public health restrictions such as the stay-at-home orders, closures of schools and non-COVID-19 healthcare services have led to adverse and unequal outcomes for some. These include increases in mental health difficulties [29], delays in cancer diagnoses [30] and reduced childhood vaccination uptake [31]. There has been a lack of return to work for some businesses that despite the financial supports were unable to remain viable. It is recognised that these impacts adversely affect the most vulnerable either by the nature of their occupation, their socioeconomic status and/or health comorbidities. In this regard, COVID-19 is being increasingly described as a “syndemic” where the virus does not act in isolation, but with accomplices such as medical disorders (cancer, heart disease, diabetes, etc.) and social factors which are interlinked and mutually reinforcing [32].

The pandemic exposed another vulnerable group of those who reside in residential care with almost two-thirds of COVID-19 deaths occurring in nursing homes [33]. Contingency plans for enhanced staffing to maintain optimal care provision will be required for future outbreaks as well as education and implementation of infection prevention and control experts [34]. COVID-19 has highlighted the need for a more integrated working relationship between the state and the residential care sector [35]. The adolescent and healthy elderly group are also recognised as vulnerable groups because of social isolations during lockdowns.

Public health measures should also consider the risk of pandemic fatigue, where initial enthusiasm and eagerness to tackle the crisis is replaced by feelings of exhaustion resulting in individuals refusing to comply with restrictions [36]. Ireland had one of the longest lockdowns in the world and strictest in the EU [37] and pandemic fatigue has been found to be associated with stricter tiered restrictions [38]. Trust in government and public health officials is crucial, however appears to be tenuous in Ireland. Three-quarters of the Irish public believed that the government’s response to COVID-19 was motivated by protecting its own reputation and the majority had more trust in the scientists than the government [39]. These concerns should be addressed to ensure effective pandemic preparedness and response agenda and scientific engagement should be maintained throughout.

The ending of the COVID-19 pandemic appears to be, in the words of T.S Elliot, “not with a bang but a whimper” [40]. Given the potential impact of the removal of government subsidies on health and occupations, government and media coverage of the decisions and potential implications would have been helpful. The opportunity for a significant debriefing of the pandemic outlining what we have learned from the COVID-19 pandemic response may have been missed. The authors welcome the upcoming report by the Public Health Reform Expert Advisory Group, chaired by Professor Hugh Brady, expected later this year [41]. This report may be the opportunity for a broader academic and social discussion around the pros and cons, benefits and adverse outcomes of the pandemic.
